# Structural and metabolic topological alterations underlying butylphthalide treatment in mild cognitive impairment due to Alzheimer’s disease and topology‐based machine learning for drug efficacy

**DOI:** 10.1002/alz.089938

**Published:** 2025-01-09

**Authors:** Xiaodong Han, Shuting Gong, Pin Wang, Ruina Li, Runqi Chen, Chang Xu, Wenxian Sun, Jin Gong, Shaoqi Li, Cuibai Wei

**Affiliations:** ^1^ Xuanwu Hospital, Capital Medical University, Beijing, Beijing China; ^2^ School of Biological Science and Medical Engineering, Beihang University, Beijing, Beijing China; ^3^ College of Integrated Traditional Chinese and Western Medicine, Changchun University of Chinese Medicine, Changchun, Jilin China

## Abstract

**Background:**

Effective early intervention of mild cognitive impairment (MCI) is the key for preventing dementia. However, there is currently no drug for MCI. As a multi‐targeted neuroprotective agent, butylphthalide has been demonstrated to repair cognition in patients with vascular cognitive impairment, and has the potential to treat MCI due to Alzheimer’s disease (AD). However, the pharmacological mechanism of butylphthalide from brain network perspective is not clear.

**Method:**

In this clinical trial, 270 patients with MCI due to AD were assigned at a ratio of 1:1 to receive either butylphthalide or placebo for one year. Alzheimer’s disease assessment scale‐cognitive section (ADAS‐cog) scores was set up as the primary endpoint, and effective treatment was defined as the decrease of ADAS‐cog>2.5. T1‐magnetic resonance imaging and fluorodexoyglucose positron emission tomography data were collected in baseline and follow‐up, and the Kullback‐Leibler divergence similarity estimation method was applied to construct the individual structural and metabolic networks. We used repeated‐measures analysis of variance to investigate group (drug group vs. placebo group; effective group vs. ineffective group)‐time interactions on ADAS‐cog and network metrics, and support vector machine was applied to develop the predictive models.

**Result:**

Butylphthalide significantly improved the cognition of patients with MCI due to AD (multivariable‐adjusted *P*<0.05). There were some overlapped structural network metrics on which both the treatment group‐time interactions and efficacy group‐time interactions were potentially significant (uncorrected multivariable‐adjusted *P*<0.05), and the metrics were: global efficiency, degree centrality and nodal efficiency of left paracentral lobule, degree centrality of right inferior temporal gyrus, and nodal efficiency of left superior frontal gyrus, medial. Of these an increased degree centrality of left paracentral lobule was significantly related to poorer cognitive improvement. Further simple effects analyses revealed an significantly increased global efficiency in structural network under both treatment and effective treatment of butylphthalide (multivariable‐adjusted *P*<0.05). Predictive model based on baseline multimodal network metrics exhibited the highest accuracy (88.93%) for predicting butylphthalide’s efficacy.

**Conclusion:**

Butylphthalide may improve cognitive function in patients with MCI due to AD by repairing abnormal organization in structural networks. Baseline multimodal network metrics could be potential predictive markers of therapeutic efficacy of anti‐AD drugs.

